# Genes Associated with Biological Nitrogen Fixation Efficiency Identified Using RNA Sequencing in Red Clover (*Trifolium pratense* L.)

**DOI:** 10.3390/life12121975

**Published:** 2022-11-25

**Authors:** David Vlk, Oldřich Trněný, Jana Řepková

**Affiliations:** 1 Department of Experimental Biology, Faculty of Sciences, Masaryk University, 611 37 Brno, Czech Republic; 2 Agricultural Research, Ltd., Zahradní 1, 664 41 Troubsko, Czech Republic

**Keywords:** transcriptome, differentially expressed gene, nodule-specific cysteine-rich peptide, gene duplication

## Abstract

Commonly studied in the context of legume–rhizobia symbiosis, biological nitrogen fixation (BNF) is a key component of the nitrogen cycle in nature. Despite its potential in plant breeding and many years of research, information is still lacking as to the regulation of hundreds of genes connected with plant–bacteria interaction, nodulation, and nitrogen fixation. Here, we compared root nodule transcriptomes of red clover (*Trifolium pratense* L.) genotypes with contrasting nitrogen fixation efficiency, and we found 491 differentially expressed genes (DEGs) between plants with high and low BNF efficiency. The annotation of genes expressed in nodules revealed more than 800 genes not yet experimentally confirmed. Among genes mediating nodule development, four nod-ule-specific cysteine-rich (NCR) peptides were confirmed in the nodule transcriptome. Gene duplication analyses revealed that genes originating from tandem and dispersed duplication are significantly over-represented among DEGs. Weighted correlation network analysis (WGCNA) organized expression profiles of the transcripts into 16 modules linked to the analyzed traits, such as nitrogen fixation efficiency or sample-specific modules. Overall, the results obtained broaden our knowledge about transcriptomic landscapes of red clover’s root nodules and shift the phenotypic description of BNF efficiency on the level of gene expression in situ.

## 1. Introduction

Legumes are plants from the family Fabaceae. Consisting of more than 750 genera and 19,500 species, this family makes up about 7% of all flowering plant species [1,2]. This widely distributed family is the third-largest flowering plant family by number of species. From an economic point of view, it is the second-most important after Poaceae (grasses). Due to their great diversity and abundance, legumes include a number of agronomic crops (grain and fodder legumes) and others serve as genetic model organisms (*Medicago truncatula*, *Lotus japonicus*) [3,4,5]. In the context of sustainable agriculture, many legume species have the potential to establish symbiosis with nitrogen-fixing bacteria and obtain access to nitrogen using biological nitrogen fixation (BNF). BNF is a process whereby plants acquire atmospheric nitrogen through interacting with bacteria capable to convert this molecular nitrogen to ammonium. This symbiotic relationship, within which the plant provides the bacteria with organic compounds used as carbon and energy source and bacteria supply the plant with fixed nitrogen, is a significant competitive advantage for plants in the occupation of nitrogen-poor soil [6].

The Fabaceae genus *Trifolium* includes more than 250 species having cosmopolitan distribution around the world [7,8], with the greatest diversity occurring in the temperate Northern Hemisphere. The economic importance of this genus relates especially to those species used extensively as fodder crops for livestock (*T. pratense*, *T. hybridum*, *T. repens*) or as a green manure plant to enhance soil fertility [9]. Due to their high content of secondary metabolites, such as isoflavonoids, some species are also being studied for potential pharmacological use [10,11,12]. Soil enrichment with nitrogen via growing plants utilizing BNF, such as clover, is more sustainable than using synthetic nitrogen-based fertilizers. However, not all genotypes within the fixation-capable species have the same nitrogen fixation efficiency [13]. Plant breeding directed to the enhancement of nitrogen-fixing ability is complicated by the complexity of this phenotypic trait, as an estimated several hundred genes are involved in the nodulation and nitrogen fixation [14,15].

The early phase of plant–bacteria interaction depends upon an early dialog between the host and microbes [16] when bacteria begin to produce their own lipochitooligosaccharide signals, termed Nod factors, in response to released plant flavonoids [17]. These signal molecules determine the specificity of the interaction itself [18,19] and are recognized by nod factor receptors on the root surface, such as NFR1 and NFR5 [20], and this causes both morphological alterations on the root surface and induction of two root-specific and one systemic pathway. While the systemic reaction, known as autoregulation of the nodulation, controls the number of nodules on the roots depending upon the nodule number already formed and regulation based on the availability of nitrogen from the soil [21], the signal pathways in the roots enable nodulation initiation and nodule formation using calcium-dependent kinases and transcription factors [22] or cytokinins [23]. 

Nodulating bacteria use infection threads to enter the root [24,25]. The bacteria then penetrate the plant cell by endocytosis as symbiosomes, which gradually differentiate into nitrogen-fixing bacteroids and further into root nodules. The nodules are specialized organs consisting of bacteroids, meristems, and vascular bundles. Nitrogen fixation is enabled by a complex of nitrogenase-nitrate reductase enzymes [26] supported by leghaemoglobin proteins located in the nodules that provide oxygen for respiratory processes into the bacteroid membrane as well as reduce the oxygen concentration inside bacteroids [27]. Two types of root nodules are distinguished: determinate and indeterminate [24]. Meristems of indeterminate nodules remain functional (genus *Medicago* or *Trifolium*); determinate nodules, however, lose their meristematic character in later stages of development (genus *Glycine* or *Phaseolus*) [28].

Indeterminate nodules are usually created by legumes belonging to the inverted repeat-lacking clade. In this clade, bacteria released into the plant cells terminally differentiate into bacteroids that cannot be cultured, show endoreduplication of their genomes, and maintain changes in the cell wall or in expression patterns [29,30,31]. Many of these changes are processed using small defensin-like peptides, especially nodule-specific cysteine-rich (NCR) peptides, which are typical for legumes with indeterminate nodules and which induce bacteroid differentiation [32]. In the best-studied legume plant, *M. truncatula*, more than 600 NCRs have been identified [33], but there are large differences in the numbers of NCR peptides among various legumes, ranging from just a few NCRs to hundreds [34].

It is estimated today that hundreds of genes with differing impacts on the phenotype are associated with the BNF process [14], and nearly 200 important genes have been identified on model legume plants using both forward and reverse genetics [35]. Originally, chemical and physical mutagens (γ-rays, ethyl methanesulfonate, fast neutron bombardment) were used to enhance the frequency of mutants and to accelerate the discovery of genes connected with BNF on such model legumes as *M. truncatula* [36,37], *L. japonicus* [38], or *Glycine max* [39]. In addition, transposon mutagenesis has broadened the possibilities for obtaining mutant populations by using Ac Transposon (*L. japonicus*; [40]), transfer DNA insertions (*L. japonicus*; [41], *M. truncatula;* [42]), retrotransposon *Tnt1* (*M. truncatula*; [43,44]) or endogenous *Lotus retrotransposon 1* [45,46] for both forward and reverse genetics. Antisense RNA/RNAi methods began contributing to a better understanding of BNF’s genetic background at the beginning of the 21st century [47,48], and over the years these have enabled the identification of many genes associated with BNF (see review by Arthikala et al. [49]). In recent years, CRISPR/Cas9 mediated genome editing has been established in legumes such as *G. max* [50], *L. japonicus* [51], *M. truncatula* [52], and *Cicer arietinum* [53] and enabled targeted mutagenesis of BNF-associated genes. Moreover, due to the current possibilities of studying genes participating in fixation, there are also papers demonstrating the advantages of using different approaches and combining methods [13,54]. In the field of synthetic biology, there are efforts to introduce nitrogen fixation into plants that have not yet been able to do so, such as cereal crops, by transferring a system of multicistronic genes connected with nitrogen fixation [55,56].

Since their development in the early 21st century, next-generation sequencing technologies have significantly accelerated genome research, identification of gene polymorphisms, and phylogenetic analyses. From that time, too, RNA sequencing has become an important and quite universal tool for transcriptome assembly, quantification of gene expression, identification of spliced variants/fusion genes, and analysis of differentially expressed genes (DEGs) [57,58,59,60]. The last of these, identifying gene expression changes between different experimental conditions or different cell populations, is the most popular application of RNA-seq to many and various questions of interest, such as in detecting genes connected with resistance against stress factors [61,62], genes regulating development [63,64], or the genes involved in a symbiotic relationship, such as BNF, where it is used for gene expression analysis in symbiotes [15,65], transcriptome profiling of nodules [66,67], and detection of expression changes during nodule development [68]. The downstream analyses of DEGs aim at functional characterization and annotation of DEGs or finding possible common patterns among them, including enrichment of certain biosynthetic pathways or Gene Ontology (GO) terms. 

The red clover genome has been de novo sequenced for the varieties *Tatra* [69] and *Milvus* [70]. In the context of BNF, Ištvánek et al. [69] identified 542 potential NCR peptides and 11 leghaemoglobin genes, and De Vega et al. [70] anchored 22,042 out of a total of 40,868 annotated genes to seven pseudomolecules and constructed a physical map enabling large-scale genomic and phylogenetic studies of traits having biological and agronomic importance. Several studies sequencing red clover transcriptomes have also been published that focus on the stress response [71,72], leaf senescence [73], splice isoforms, fusion gene and non-coding RNA [74,75], and leaf variegation [76]. Owing to the complexity of this trait, and even though red clover has a high level of BNF heritability [77], phenotypic-level understanding of nitrogen fixation is insufficient. Trněný et al. [13] identified candidate genes associated with BNF efficiency as well as polymorphisms associated with BNF and reflecting phenotype variability. Our knowledge of the genetic variation within BNF must be expanded on the level of gene expression and transcriptomic analysis. 

The goals of our experiments, therefore, were to obtain red clover populations with different levels of nitrogen fixation and perform differential gene expression analysis using RNA sequencing of root nodules of red clover genotypes with contrasting nitrogen fixation levels. The annotation of differentially expressed genes between genotypes with high and low nitrogen fixation efficiency was directed to finding their functions and thereby allowing their connection with biosynthetic pathways associated with BNF. NCR peptides in nodule transcripts were identified and characterized to evaluate their connection to BNF efficiency, and evolutionary analyses were aimed at revealing the roles of different modes of duplicated genes in BNF.

## 2. Materials and Methods

### 2.1. Plant Materials and Growth Conditions

In total, 378 genotypes of two diploid (*Start*, *Global*) and three tetraploid (*Tatra*, *Tempus*, *Kvarta*) *T. pratense* varieties were grown in 2019. These genotypes were the progeny of 16 plants (8 with high and 8 with low BNF efficiency) evaluated in 2017 [13]. This progeny was used to observe the selection for nitrogen capacity in field conditions.

The red clover seeds were scarified and germinated on wet perlite. Germinated seeds were then sown in individual pots filled with perlite and inoculated with rhizobia by adding 1 mL of *Rhizobium leguminosarum* bv. *trifolii* inoculum provided by the Crop Research Institute (Prague, Czech Republic). Different rhizobia strains were used for diploid and tetraploid varieties according to the recommendations of the collection’s curator. Plants were grown in a greenhouse within individual pots filled with perlite regularly watered with nitrogen-free nutrient solution as described earlier [13]. Before evaluating nitrogen fixation efficiency, the fresh mass of the shoots and roots of analyzed plants was measured in milligrams using analytical balances after pulling them out of the pots and removing perlite. 

### 2.2. Evaluation of Nitrogen Fixation Efficiency, Sample Preparation, and RNA-Sequencing

Nitrogen fixation efficiency was evaluated by acetylene reduction assay (ARA) while measuring nitrogenase activity in individual plants approximately 100 days after sowing [78]. ARA was performed on the sheared roots with nodules placed in a jar with added acetylene on a total of 378 plants. The results were expressed as ethylene molar concentration (µmol/mL) in a jar after 0.5 h of incubation using equations according to Unkovich et al. [79]. The ethylene level was related to particular plants as we assessed whole genotypes. After ARA, the roots were again planted in pots to let the plants regenerate, and root nodules were sheared from chosen genotypes 14 days after ARA. Eight red clover genotypes chosen for RNA sequencing were represented by two contrasting groups according to their BNF rates (low × high BNF efficiency) and by two diploid and two tetraploid plants in both groups. Three biological replicates were collected from each genotype, and 20–30 mg of root nodules were flash-frozen in liquid nitrogen for each replicate.

RNA isolation was performed for 24 samples chosen for RNA sequencing (3 biological replicates for each of the 8 chosen genotypes) using RNeasy Plant Mini Kit (Qiagen, Hilden, Germany) according to the manufacturer’s protocol, and DNase treatment was performed using TURBO DNA-free^TM^ Kit (Invitrogen/Thermo Fisher Scientific, Waltham, MA, USA). RNA integrity was checked on a 1.2% agarose gel and fragment analyzer system (Agilent, Santa Clara, CA, USA), and RNA concentration was quantified by the Nanodrop 2000c spectrophotometer (Thermo Fisher Scientific, Waltham, MA, USA). Library preparation and RNA sequencing were performed at the Genomics Core Facility of CEITEC MU (Brno, Czech Republic). RNA-seq library was prepared from total RNA using poly(A) enrichment of the mRNA with NEBNext^®^ Ultra™ II Directional RNA Library Prep Kit for Illumina (New England Biolabs, Ipswich, MA, USA), and the library was sequenced for 75-bp reads with paired-end sequencing on an Illumina NextSeq 500 instrument (San Diego, CA, USA) using the NextSeq 500 High Output Kit.

### 2.3. Bioinformatic Analysis

The basic characteristics of the obtained reads were checked in FastQC v0.10.1 [80]. Reads were qualitatively filtered, contaminant-filtered, trimmed using scripts filterbytile.sh and bbduk.sh, which are part of the BBmap scripts [81], and then aligned onto the reference genome of *T. pratense* variety *Milvus* [70] using the STAR aligner [82] and SAMtools [83] for BAM file indexing. Quality control of aligned reads was performed with QoRT [84] while using gffread, part of the Cufflinks package [85], for gff3/gtf format conversion. Aligned reads were quantified using gene-based read counting with FeatureCounts [86]. Normalizing read counts and DEG analysis were performed using the DESeq2 package in R [87] along with RStudio [88]. Prior to DEG analysis, the raw read counts were first normalized for sequencing depth differences using DESeq2 size factor and log2 transformed; the similarity of gene expression patterns in biological replicates was checked using the Pearson correlation coefficient (*r*), hierarchical clustering (distance measure d = 1 − r; complete linkage) and principal component analysis (PCA). The following DEG analysis evaluated contrast among BNF samples, with low BNF set as the default state. Genes with log2 fold change >1 and adjusted *p*-value < 0.05 were considered as differentially expressed.

DEGs were annotated using the reference annotation file [70] extracted from Phytozome [89] and the LegumeIP database [90]. Unannotated DEGs were further functionally annotated using blastp ver. 2.6.0+ [91] (e-value 1–10) against several databases: TrEMBL and Swiss-Prot [92], all predicted proteins from Phytozome [89], the annotated files from *T. pratense* variety *Tatra* [69] and from *T. subterraneum* [93]. For each analyzed sequence, the hit with the highest score and the lowest e-value was chosen as an annotation. The custom Python scripts were used for filtering, extracting, and merging data during annotation. Annotation terms (Gene Ontology [GO], Kegg orthology [KO]) were assigned to proteins by manually transferring the terms from Swiss-Prot or Phytozome. Furthermore, the Blast2Go pipeline [94] in OmicsBox [95] was used for improving the functional annotation of DEGs. Using Blast2GO, protein sequences of DEGs were queried against the NCBI non-redundant protein sequences using blastp (e-value 10^–3^). InterProScan in Blast2Go was carried out to retrieve the domains and motifs. GO terms connected with the obtained BLAST hits were retrieved and GO annotation was performed with Blast2GO (e-value hit filter: 10^−6^). Corresponding GO terms associated with InterProScan results were transferred to the sequences and merged with already existing GO terms. Finally, KEGG (Kyoto Encyclopedia of Genes and Genomes) pathway analysis and GO enrichment (*padj* Benjamini-Hochberg < 0.05) were carried out using Blast2Go.

Genes without experimental evidence (unconfirmed) were identified by comparing original annotation files from De Vega et al. [70] and annotation of *T. pratense* downloaded from Phytozome [89]. (Phytozome excluded any gene without experimental evidence). Extracted unconfirmed genes were annotated as described above.

### 2.4. Experimental Verification of Sequencing Data

Verification of acquired sequencing data was performed using quantitative polymerase chain reaction (qPCR). Ten genes with detectable expression according to RNA-seq were randomly chosen for qPCR. Primers were designed using the Primer3 tool (http://primer3.ut.ee, accessed on 28 October 2021; [96]); their specificity was checked using Blast+ (ver. 2.8.1; https://blast.ncbi.nlm.nih.gov/Blast.cgi, accessed on 28 October 2021; [97]) with the *T. pratense Milvus* genome as a database [70]. RNA samples were isolated, and DNase treated as described above, reverse transcription was performed using a High Capacity cDNA Reverse Transcription Kit (Applied Biosystems/Thermofisher, Foster City, CA, USA). Approximately 2 µg of prepared cDNA was taken as the template for qPCR using SYBR^®^ Select Master Mix (Applied Biosystems) and primer pairs shown in Supplementary Table S1. Cycling conditions were set as follows: 2 min at 50 °C, 2 min at 95 °C followed by 40 cycles of 3 s at 95 °C and 30 s at 60 °C. The *UBQ* (ubiquitin) gene was chosen as the reference gene. Sample cycle threshold (Ct) values were standardized for each template using the reference gene. The sample with the lowest expression was used as a calibrator and the 2^−ΔΔCt^ method was used to analyze the relative changes in gene expression. Three replicates per sample were used to ensure statistical credibility.

### 2.5. Identification of NCR Peptides in T. pratense

Protein sequences encoded by genes detected in nodules across all analyzed samples were inspected using a custom Python script searching conservative NCR-like structure according to Maróti et al. [98] and length < 150 bp. Sequences identified as NCR-like were merged with those detected as DE. Then, NCR-like sequences were analyzed using blastp ver 2.6.0+ (e-value 10^–5^) [97] against the non-redundant protein sequences database [99] and conserved regions were detected using NCBI Conserved Domains Database [100]. Signal peptides were searched using the SignalP tool [101], and subcellular localization was identified using DeepLoc [102]. Physicochemical parameters were computed using ProtParam [103], and 3D structures were predicted using Phyre2 [104] and trRosetta [105].

### 2.6. Gene Duplication Analyses

Different modes of gene duplication in *T. pratense* were identified using Plant Duplicate Gene Database (PlantDGD [106]). The custom Python scripts were used for filtering, extracting, and merging PlantDGD data with RNA-seq results. In this way, each gene expressed in the nodule was inspected for possible duplication events and each gene was classified according to its own duplication mode (whole genome duplication [WGD], tandem, proximal, transposed, dispersed, non-duplicated). The distribution of different duplication modes was checked among DEGs, and this distribution was compared with the global distribution of duplicate modes among all genes expressed in nodules. Statistical significance was calculated using Pearson’s *chi*-squared test for global distribution and Fisher’s exact test for particular duplicate modes in R while using RStudio [88].

The expression diversity between duplicated pairs was computed for those duplicated pairs in which at least one gene copy was identified as differentially expressed. The Pearson correlation coefficients (*r*) between the expression profiles of analyzed gene pairs were calculated using Python’s Numpy module. First, a cut-off *r*-value was determined below which two duplicated gene pairs were considered divergent in expression. Then, 10,000 gene pairs were randomly selected and *r*-values for their expression profiles were calculated. Overall, 95% of the *r*-values for randomly chosen gene pairs were *r* < 0.67. That means the gene pairs with *r* ≥ 0.67 have significantly conserved expression levels at *α* = 0.05. Therefore, the gene pairs with *r* < 0.67 were considered to have diverged in expression.

### 2.7. Expression Network WGCNA Analysis

Counts of genes were transformed by variance stabilizing transformation using the DESeq2 R package [87]. Data normalization was also part of the transformation. For further WGCNA analysis [107], we filtered out genes having occurred in less than 3 samples with transformed counts greater than or equal to 10. From a total 40,868 genes, 25,873 genes met these criteria and were used for WGCNA analysis. The adjacency matrix is based upon a topological overlap matrix of “signed” type. Modules were identified in one block using soft threshold power = 6, network type “signed hybrid”, minModuleSize = 20, mergeCutHeight = 0.45, and deepSplit = 3. A gene co-expression network was drawn using Cytoscape 3.9.0 with edge included by adjacency threshold = 2 [108]. GO enrichment testing was performed by hypergeometric test using the BiNGO 3.0.3 app of Cytoscape [109]. Multiple testing correction was made using Benjamini Hochberg FDR [110]. Considered as enriched were those GO terms having false discovery rate (FDR) *p*-values of enrichment test less than 0.05. The functional content of GO enrichment terms of correlated groups was summarized through clustering of GO terms of genes within modules using GOMCL, a toolkit to cluster, evaluate, and extract non-redundant associations of GO-based functions [111], according to default parameters.

## 3. Results

### 3.1. BNF Efficiency Measurement 

**Figure 1:**
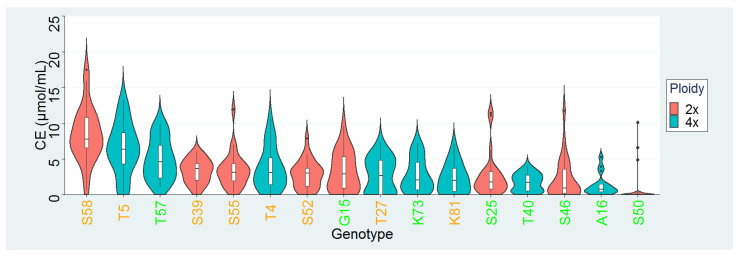
Intrapopulation distribution of BNF efficiency among accessions evaluated prior to RNA sequencing. Varieties indicated by letter: A–*Tatra*, K–*Kvarta*, S–*Start*, T–*Tempus*, G–*Global*. Numerals following the letter indicate number of the parent plant from 2017. Inside the violin plots, median and interquartile ranges indicated by boxplot, minimum and maximum by whiskers, outliers by dots above boxplot. Accessions are ordered by median values of nitrogen fixation. Orange labels–progeny of strong fixators from 2017, green label–progeny of weak fixators from 2017. On the y-axis, measured BNF efficiency is expressed as concentration of ethylene in µmol/mL.

Overall, 378 genotypes of the 16 parental plants of two diploid and three tetraploid T. pratense varieties were evaluated using ARA. The characteristics of intrapopulation diversity of BNF efficiency among accessions are demonstrated in Figure 1 (partially published in Trněný et al. [13]).

Figure 1 shows that outliers with several times greater BNF efficiency compared to others exist in several populations. The interpopulation variability was high because plants are progeny of either strong- or weak-fixing parental plants and thus influenced by selection. The distribution of intrapopulation fixation level differed slightly between strongly and weakly fixing varieties. In weak-fixing populations, the largest proportion of plants had low fixation efficiency ranging from near zero and up to the mean level, while a smaller part of genotypes had higher efficiency. In strongly fixing populations, the largest proportion of plants had fixation efficiency around the mean level and these proportions decreased to both sides from the mean. Generally, the progeny of plants with high BNF efficiency from 2017 datasets (orange label) had significantly higher BNF efficiency than did the progeny of plants with low nitrogen fixation (green label) (one-tailed Mann–Whitney U test; *p* = 2.528 × 10^−8^). 

**Figure 2:**
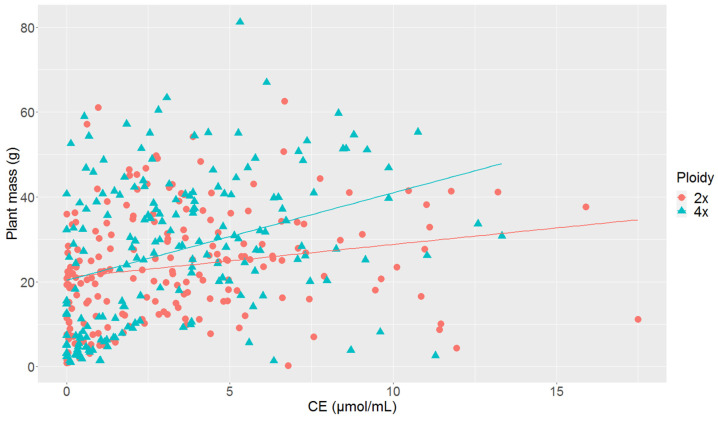
Dependence of measured BNF efficiency expressed as ethylene molar concentration (CE) on plant fresh mass with linear regression lines. Ethylene molar concentration is expressed as ethylene concentration in µmol/mL.

Together with ARA evaluation, analyzed plants also had been measured for their plant fresh mass (Figure 2). The analyzed plants were planted with no exogenously supplied nitrogen, which means that nitrogen availability was one of the crucial factors influencing plant growth and the plants were forced to obtain nitrogen using BNF under these conditions. Figure 2 demonstrates that plants with higher BNF efficiency tended to achieve greater fresh mass and this tendency was clearer in tetraploid plants.

### 3.2. Differential Gene Expression Analysis

As sequenced on the Illumina NextSeq 500 instrument, the transcriptome of 24 samples represented four accessions with high BNF efficiency and four accessions with low BNF efficiency in biological triplicates selected by ARA. Diploid accessions with high/low BNF efficiency were S46/12, S46/7, S55/6, and S25/17. Tetraploid accessions with high/low BNF efficiency were A16/17, A16/21, T57/20 and T57/22. One of the twenty-four samples was poorly sequenced due to an unidentified problem during sequencing, and this sample was omitted from the following analysis. From the total 23 samples analyzed, 428.9 million pair-end reads were assigned to the samples with an average of 18.6 million per sample (Supplementary Figure S1), and the total output was 65.2 Gb. Barcodes were not recognized in 27.1 million reads and these samples were omitted from the analysis.

After mapping sequencing reads to the reference genome, alignment control was performed to evaluate the mapping rate and quality of the sequencing reads. In general, the number of uniquely mapped reads was about 80%, as shown in Supplementary Figure S1, and gene-body coverage showed a general abundance of reads across transcript bodies to be equal, with a median of around 50 and thus showing no possible 3′ or 5′ biases. 

Prior to DEG analysis, the similarity of gene expression patterns of biological replicates was checked using Pearson correlation coefficient (r), hierarchical clustering, and PCA. Pearson correlation coefficients of rlog normalized biological replicates’ r-values were >0.99 in seven out of eight biological replicates (Supplementary Figure S2). A dendrogram of their hierarchical clustering is shown in Supplementary Figure S3. The individual biological replicates are well separated, and the accessions with low and high BNF form separate groups in the dendrogram. Similarly, the PCA plot (Supplementary Figure S4) shows those accessions with different levels of nitrogen fixation to be well separated from one another and that biological replicates are grouped together.

**Figure 3:**
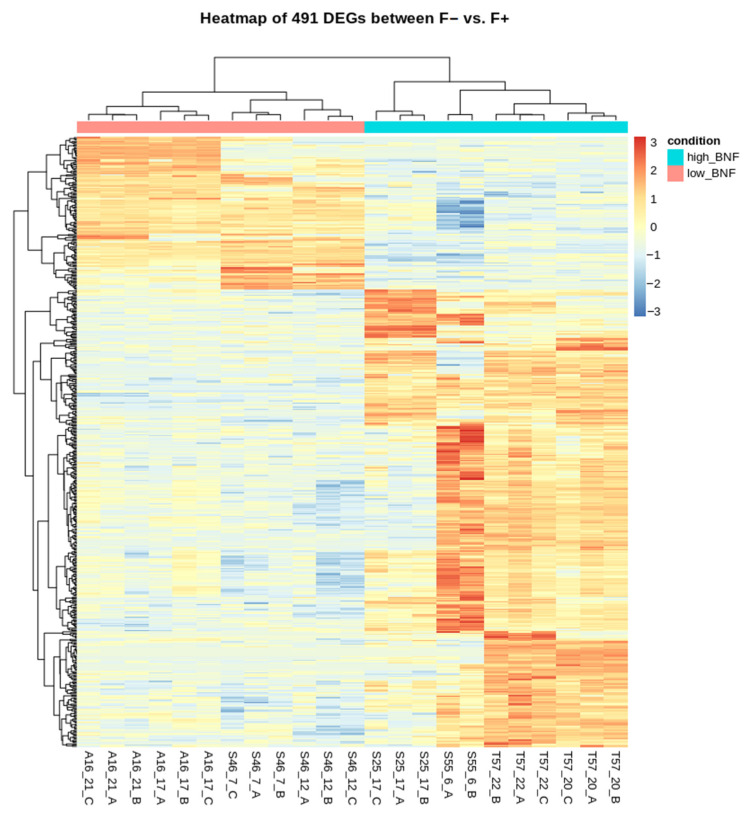
Heatmap of rlog-transformed read counts for 491 differentially expressed genes. Genes are sorted according to hierarchical clustering and the read count values are scaled per row. High-BNF samples are on the right side of the plot (blue line), and low BNF are on the left (red line).

**Figure 4:**
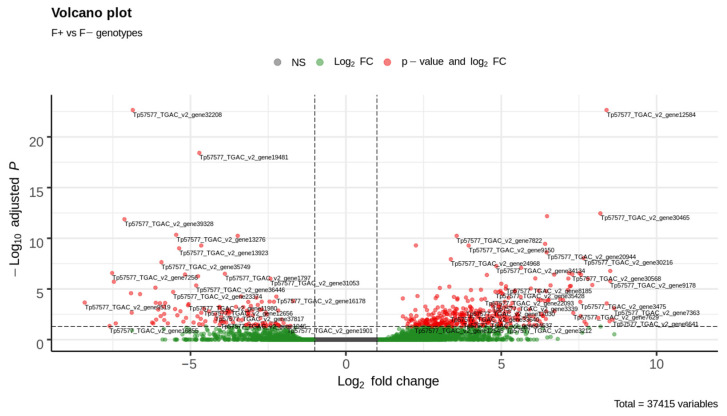
Volcano plot for 491 differentially expressed genes (red dots). The vertical dashed line indicates threshold log_2_ fold change > 1, the horizontal dashed line indicates threshold *padj* > 0.05.

DEG analysis was performed between sequenced contrasting BNF accessions and low BNF was set as the default state. As a result, 37,415 expressed genes were revealed in the sequenced nodules, and 8713 genes (23%) were low counts with average expression < 2 reads. Overall, 491 genes were identified as differentially expressed (log_2_ fold change > 1, padj < 0.05 were set as thresholds) and 368 genes were overexpressed compared to low-BNF accessions while 123 genes were underexpressed (Figure 3 and Figure 4). A global view of the relationship between expression change and average expression strength is demonstrated by the MA plot in Supplementary Figure S5. The list of DEGs is attached in Supplementary Table S2.

### 3.3. Annotation of Differentially Expressed Genes

DEGs were first annotated using an annotation file from sequencing of the *Milvus* variety of *T. pratense* [70]. Of 491 DEGs, 375 (76%) were assigned to at least one category of functional annotation (Pfam, PANTHER, KOG, ec, KO, GO) and the remaining 116 genes were without any hit. Of 116 unannotated genes from the reference annotation file, 71 were partly annotated for at least one of the aforementioned categories using publicly available databases as described in the Methods. Functional annotation was further improved using Blast2Go, and the same pipeline was used for GO enrichment and the inclusion of DEGs in biosynthetic pathways (Table 1). Finally, 446 of 491 DEGs (91%) were successfully annotated for at least one of the functional annotation categories (Pfam, PANTHER, KOG, ec, KO, GO). 

### 3.4. Experimental Evidence of Unconfirmed Genes 

The original annotation file of *T. pratense* variant *Milvus* [70] used for DEGs contains genes both experimentally confirmed and unconfirmed during assembly and annotation. Unconfirmed genes are those for which the authors could find no transcripts supporting these genes using RNA-seq. During the annotation of 37,415 genes detected in nodules, 863 of these were originally unconfirmed. The expression of the most abundant unconfirmed gene was more than 13,000 mapped reads, 235 genes were considered as low counts with average expression < 2 reads, and 11 of these 863 genes were identified as DE. The expression distribution of the originally unconfirmed genes is demonstrated in Supplementary Table S3.

Of these 863 genes, 596 (69%) were at least partly annotated using GO terms and 690 of them (80%) had at least one hit using InterProScan. The most frequently appearing GO bp terms in the originally unconfirmed genes are listed in Supplementary Table S4. The list of originally unconfirmed genes is attached in Supplementary Table S5.

### 3.5. Experimental Verification of Sequencing Data

**Figure 5:**
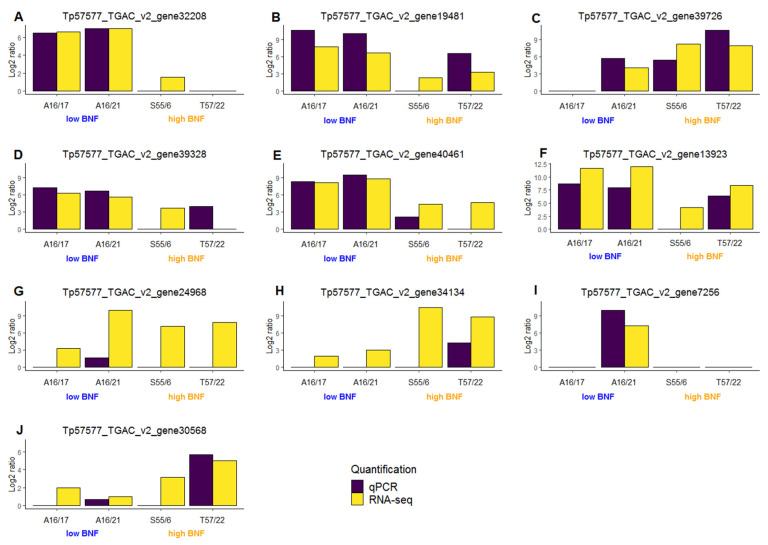
Verification of sequencing data using qPCR for 10 genes (subfigures **A**–**J**) in *T. pratense* genotypes with low and high BNF. All samples were analyzed in triplicate and data are presented as means. The *x*-axis identifies analyzed samples, and the *y*-axis shows relative expression in the log_2_ ratio.

Ten DEGs with detectable expression according to RNA-seq were verified using qPCR on both high and low-nitrogen fixing samples of *T. pratense*. *UBQ* was selected as the reference gene because it showed the most stable expression across different *T. pratense* samples during analogous experiments. For each analyzed gene, primer pairs (Supplementary Table S1) were functional and created a detectable fluorescence signal in at least one of the analyzed samples. The expressions of the genes analyzed using qPCR were in agreement with those from the RNA-seq (Figure 5A–J). Panels A–F and J in Figure 5 show a very good correlation in expression between RNA-seq and qPCR. Panels G and H in Figure 5 show weak expression in qPCR (high Ct value and low fluorescence) that hinders verification while panel I shows that only one genotype had a detectable expression of the analyzed gene for both RNA-seq and qPCR.

### 3.6. Identification of NCR Peptides

Protein sequences of 37,415 genes detected in nodules across all samples were inspected for NCR-like structure [98]. This structure, with four or six conserved cysteines, was found in 33 sequences with a length of <150 bp. Four of these were differentially expressed between high and low nitrogen fixing samples while thirteen were marked as low counts and were omitted from the DEG analysis. The sequences were annotated using BLAST and analyzed for signal peptides, subcellular localization, conserved amino acids and domains, and physicochemical parameters to confirm or exclude their inclusion into the NCR peptides. Finally, 4 sequences out of the 33 *T. pratense* sequences detected in nodules with NCR-like structure and length >150 bp were confirmed as NCR peptides (Table 2). The others were either unconfirmed or ruled out as NCR peptides using subsequent in silico analyses.

### 3.7. Gene Duplication Analysis within Differentially Expressed Genes

Four hundred ninety-one genes identified as differentially expressed between samples with high and low BNF samples were inspected for possible duplication events and were classified according to a duplication mode (whole genome duplication [WGD], tandem, proximal, transposed, dispersed, non-duplicated). Similarly, the distribution of different modes of gene duplication was inspected across all genes expressed in nodules. The distribution of different modes of gene duplication differs among DEGs and all genes expressed in nodules (Χ-squared = 24.108, *df* = 5, *p*-value = 0.0002): The numbers of genes originated from tandem and dispersed duplication were significantly higher in DEGs while the genes originated from transposed duplication and non-duplicated genes were under-represented in DEGs (Table 3).

**Figure 6:**
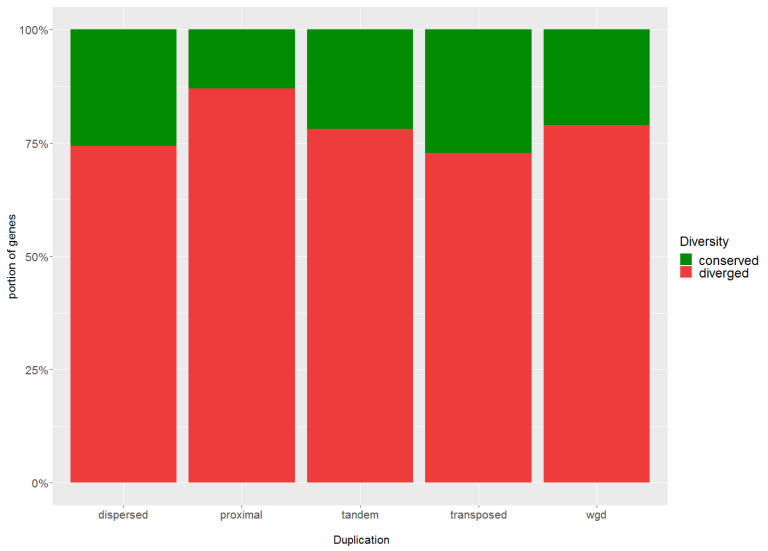
Expression divergence between duplicated gene pairs originated from different modes of duplication. Duplicated pairs were divided according to mode of duplication (*x*-axis) and proportions of genes with conserved versus diverged expression were calculated (*y*-axis).

For estimating expression divergence, the Pearson correlation coefficients (*r*) among the expression profiles of the analyzed genes were calculated for those duplicated pairs within which at least one gene copy was identified as differentially expressed. A cutoff *r*-value below which two duplicated gene pairs were considered divergent in expression was determined. Inasmuch as 95% of the *r*-values for 10,000 randomly chosen gene pairs were <0.67, those gene pairs with *r* < 0.67 were considered to have diverged in expression at *α* = 0.05. The duplicated gene pairs were divided according to the mode of duplication and the expression diversity was inspected separately for each duplication mode. Overall, 72–79% of duplicated pairs from dispersed, tandem, transposed, and WGD duplication diverged in expression, and a higher proportion (87%) of duplicated pairs with diverged expression was evaluated for those originated from proximal duplication (Figure 6).

### 3.8. Weighted Correlation Network Analysis

**Figure 7:**
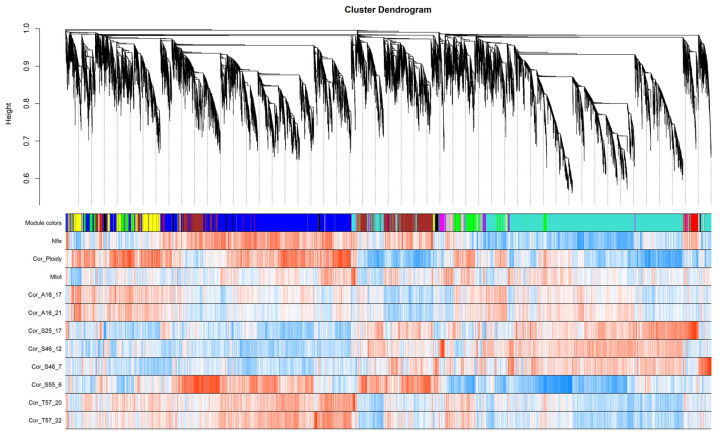
WGCNA analysis: Clustering of genes according to their expression profile similarity. First row assigned a module color to each gene. Rows 2 to 12 are red–blue scale heatmaps of Pearson correlation coefficients between traits and expression levels of the genes (red: 1, blue: −1).

WGCNA identified 16 modules, each containing genes with similar expression profiles in all samples analyzed. The distribution of genes into modules and their clustering according to similar expression profiles can be seen in Figure 7. The counts of genes in each module are in Table 4. The two most common modules are Turquoise and Blue, with 9097 and 7178 transcripts, respectively. These two modules are characterized by near mirror-image expression profiles between the two sample groups (samples A16_17, A16_21, S25_17, S46_12 and S46_7 vs. samples S55_6, T57_20 and T57_22). Eigengenes for each module were determined to reflect the common expression trend for genes belonging to that module (Supplementary Figure S6). Eigengenes are defined as the first principal component of each module and represent the module expression profile. A network (Supplementary Figure S7) was created to illustrate the relationships among gene expression profiles and modules. The network shows a pattern of two large groups of genes that are linked together by the Brown module genes. The first group is mainly composed of the Blue, Yellow, and Brown module genes. The second large group is made up of genes of the Green, Turquoise, Red, and Purple modules. 

The eigenvalues of the modules found were further correlated with the descriptive traits and the correlation coefficients were plotted in heatmaps (Supplementary Figure S8). The correlation criterion was determined by a *p*-value < 0.01. The expression profile of a given module is represented by its module eigenvalues which we can correlate with a specific trait. We designed 16 modules, half of which correlate with genotype-specific modules (Midnightblue, Red, Magenta, Tan, Brown, Green, Cyan, Grey). The eigengenes of the Red, Blue, and Salmon modules were most positively correlated with the nitrogen fixation level. Conversely, the Greenyellow, Purple, and Turquoise modules were negatively correlated with nitrogen fixation. The modules Green, Yellow and Blue were negatively correlated, and the Brown module was the most strongly correlated positively with the ploidy level. The Cyan module is correlated the most positively with the sample weight trait and the Grey module is correlated most negatively with that trait (Supplementary Figure S8). For groups of genes in all modules positively or negatively correlated with nitrogen fixation level, ploidy, and weight, and for groups of DEGs GO annotation, enrichment test and GOMCL summary were performed (Supplementary Table S6).

## 4. Discussion

The *Rhizobium*–legumes symbiosis has received much attention in recent decades because soil enrichment by nitrogen using BNF has environmental and ecological advantages over the use of synthetic nitrogen fertilizers. Realization of this phenotypic trait, however, is facilitated by the interaction of two genomes (plant × bacteria) along with an influence of the environment. These conditions, taken together with the involvement of hundreds of genes connected with nodulation and nitrogen fixation, impede research into BNF and the identification of genes with a major influence on BNF efficiency and their utilization for agronomic purposes [14,35]. Therefore, the amount of fixed nitrogen acquired by current nitrogen-fixing plants is far below its potential. It has been estimated that the amounts of fixed nitrogen could be increased by as much as 300% through plant breeding and utilizing genotypes highly efficient in BNF [112]. Moreover, BNF efficiency is a highly variable trait differing not only between species [113] but also among individuals within a given species [13]. 

Due to the estimated high broad-sense heritability of this trait in relatively stable field conditions (more than 0.8 in *G. max* [114] and 0.9 in inbred lines of *T. incarnatum* [115]), the potential for selecting highly effective BNF genotypes is high, although it has been reported that efficiency of particular genotypes is greatly influenced by both environmental conditions (soil acidity, phosphorus availability) and symbiotic partner [116,117,118]. That was the reason why we followed up on the conclusions of Trněný et al. [13]; we evaluated the BNF efficiency in the next generation of strong- and weak-fixing red clover genotypes analyzed and evaluated in their publication. Because there is not a consistent opinion regarding the effect of ploidy upon BNF [119,120], diploid and tetraploid red clover genotypes of different red clover varieties were equally included in both contrasting groups (strong and weak fixators) to minimize the effect of ploidy upon BNF efficiency, and all analyzed plants were planted and maintained under the same conditions to reduce the environmental effect. 

Among several methods developed for assessing BNF [79], ARA is one of the most widespread and is favored for its high sensitivity, and high throughput potential, especially for comparative purposes in manipulative experiments [121]. Because many factors influence the measured BNF rate, such as temperature [122], light [123], ecosystem successional stage [124] or seasonal/diurnal variations [125,126], ARA is less suitable for obtaining absolute values. As in our case, however, uniform measurement conditions at a specific time enable acquiring relative rates of BNF [127] and thus ARA was a method well suited to our purposes. 

RNA-seq and the following differential gene expression analysis were focused upon the discovery of genes differentially expressed to a statistically significant extent within nodules between genotypes with high and low BNF efficiency regardless of ploidy and red clover variety and while controlling for effects of environmental conditions. Nodules served as the target tissue for evaluating nitrogen fixation. The expression profiles obtained reflected the involvement of plentiful genes for processes such as legume–rhizobia interaction and nodule development, and almost 500 DEGs were identified from RNA-Seq data. For the first time, our results report the assessment of genes influencing the efficiency of BNF in red clover.

Because a number of genes were annotated not at all or only in part, annotation of DEGs across genotypes was a necessary step to find their functions and allow their connection to biosynthetic pathways. Insomuch as red clover is not a model genetic plant, its first genome assembly was published only in 2014 [69], nine years later than the draft genome sequences of legumes *M. truncatula* and *L. japonicus* [128]. One year later, another assembly was published [70] together with the construction of a physical map. Although we used both available annotations to decipher the functions of DEGs, this approach was not sufficient because about a quarter of the DEGs were without any annotation, thereby hindering the disclosure of their functions. Thus, we attempted to improve annotation using recently published annotation files of closely related species. This approach helped to improve annotation and allowed at least one functional annotation category to be assigned to each of more than 90% of DEGs. Even improved annotation, however, is not sufficient to identify the functions of many genes detected as DE, and limited assignment to some functional annotation category may merely suggest rather than reveal a possible function. 

As a result of our analyses, DEGs encoded the highest number of enzymes as associated with sesquiterpenoid and triterpenoid synthesis. Terpenoids constitute a highly diverse and widely distributed group of secondary metabolites in plants playing various roles in plant defense, determination of membrane fluidity, or plant growth [129,130,131]. In the context of BNF and nodulation, it has been demonstrated that terpenoids are able to induce the expression of Nod factors or genes involved in the Nod signaling pathway [132]. Moreover, strigolactones, a group of terpenoid lactones acting as hormones, exhibit various roles in root growth and formation of root nodules in legumes [133,134], and strigolactone genes influence nodulation by inducing the expression of Nod factors of rhizobial bacteria [135]. Among other enriched pathways, several “sugar-related” signaling pathways were found: pentose and glucuronate interconversions (PGI), starch and sucrose metabolism (SSM), galactose metabolism (GM), and amino sugar and nucleotide sugar metabolism (ASNSM). Akbar et al. report the activation of PGI and SSM pathways during salt stress in cotton [136], and those authors hypothesized that the modification of these pathways could lead to significant tolerance to the salt stress. Similarly, shifting concentrations of metabolites within the PGI pathway were found during a study of stress response and host defense against plant herbivores [137]. GM and ASNSM pathways are well-studied in fungal pathogens or pathogen–plant interactions because the metabolites of these pathways are utilized on the wall surfaces as compounds of fungal and/or plant cell walls or virulence factors [138,139,140]. Among enriched pathways was also phenylpropanoid biosynthesis. Metabolites of this pathway then enter into multiple other pathways, such as lignin and flavonoid biosynthesis, and contribute to the response to both biotic and abiotic stimuli. They are indicators of various stress factors and mediators of particular stress tolerance [141]. They help to invade new habitats [142], or they influence the stability or robustness of plants in relation to mechanical or environmental factors such as drought using phenylpropanoid-based polymers [143]. Flavonoids, secondary metabolites of one of the branches of the phenylpropanoid pathway, are known to have multiple roles during the processes of nodulation and nitrogen fixation. They act as signal molecules during the early phases of the rhizobia and plant interaction [144] or serve as polar auxin transport inhibitors leading to nodule organogenesis [145].

Taken together, the pathways enriched by the representation of DEGs encoding particular enzymes are directly connected with nodulation and BNF (terpenoids, flavonoids), and the metabolites of the others can influence the BNF performance through several possible effects. For instance, metabolites of enriched “sugar-related” pathways are reported to have shifts in concentration under various stress conditions, thus indicating that these compounds could be involved in mechanisms for stress response. Although colonization of symbiotic rhizobia usually does not elicit plant defense mechanisms [146,147], the particular step during nodulation could be a cause of defense response under some circumstances because a plant controls every aspect of the correct nodulation process. In case of any problem or defect, the defense response can occur, and a plant can undergo some sort of stress condition. That means that the enrichment of pathways more or less connected with stress responses between genotypes with high and low fixing efficiency could result from the fact that the process of nodulation has not developed correctly, probably in weak-fixing genotypes, and resulting in plant stress response. Alternatively, some genotypes could have undergone some type of stress conditions (e.g., infection, mechanical damage) before they were analyzed, although all plants were planted and maintained in the same way, and these stress stimuli could have an effect on nodulation and BNF efficiency. Table 5 summarizes the top 10 enriched GO bp terms, and stress response is one of the most enriched. That supports this hypothesis. Other enriched GO bp terms include several responses to stimulus, developmental processes, or interaction with different organisms, all of which are terms relating to biological processes expected in the context of legume symbiosis and nodulation.

Leghaemoglobin genes were among the genes with the highest expression in the nodule transcriptome (Table 6). The same finding has been proven in *M. truncatula*, where genes for leghaemoglobin were also among the most strongly expressed genes in nodule transcriptome [66], and both species, too, have similar numbers of leghaemoglobin genes [69]. Leghaemoglobin proteins are necessary for the activity of the enzyme nitrogenase [148]. Because nitrogenase is irreversibly inactivated by oxygen [149], leghaemoglobins reduce free oxygen levels inside the bacteroids while allowing ATP production by transporting oxygen for respiratory processes on the bacteroid membrane [150].

Inasmuch as red clover has nodules of an indeterminate type whose bacteroids are terminally differentiated, NCR peptides play an important role in nodule development, especially in bacteroid differentiation. Therefore, we strove to identify NCR peptides expressed in nodule transcripts and evaluate their predicted functions using in silico approaches. Ištvánek et al. [69] predicted 542 genes for NCR peptides during the first red clover assembly using tblastx searches against NCR peptides of *M. truncatula*, and that number is comparable with those identified in this model legume [151]. In contrast to this prediction, we were able to identify only 33 genes within the nodule transcriptome that met the criteria set for the search for genes encoding NCR peptides (structure, conserved cysteines, length). Only 33 out of 37,000 genes detected in nodes had a conserved structure with 4 or 6 cysteines and length <150 bp, and for only 4 out of these 33 sequences were their functions supported by in silico analysis assessing, for example, signal sequence or subcellular location and BLAST searches. These differences in amounts of predicted and detected NCR peptides arose mostly due to our use of different methods. To predict NCR peptides, BLAST searches were performed regardless of structure, length, or other aspects that were considered in identifying NCR peptides in this study. Moreover, not all similar genes need to be really NCR in nature. They can be pseudogenes or can have different functions, such as producing defensins instead of functioning in root nodule symbiosis. As a result, many genes predicted as NCR during red clover assembly lack the typical NCR structure with conserved cysteines. The resulting number of sequences found was significantly lower compared to those in *M. truncatula*, but the abundance of NCR peptides among legume species has been reported to be highly variable [34]. The high number of NCR peptides can be due to: (1) constrained rhizobial growth in nodules, (2) selection against cheaters, (3) control of bacteroid development and metabolism, or (4) a combination of these points. Lower numbers of NCR peptides have been identified in several other legumes, such as 63 in chickpea (*C. arietinum*) [152] and 7 in *Glycyrrhiza uralensis* [32]. 

Gene duplication is considered to be one of the most important evolutionary mechanisms generating plentiful raw materials for processes such as speciation or neofunctionalization [153]. Gene duplication was realized by several mechanisms to varying degrees that include, among others, single gene duplication and whole genome duplication. Single gene duplication consists of four types: tandem (TD), proximal (PD), transposed (RD), and dispersed duplication (DSD) [106]. In the context of BNF, WGD has been extensively studied in connection with an ancient polyploidy event that occurred in a Papilionoideae lineage of legumes approximately 58 Ma ago [151]. Although it is generally supposed that this event did not precede BNF, it might have facilitated and refined the BNF system using genetic materials provided by this polyploidy event [154]. Here, we classified DEGs into five groups according to the duplication mode. We inspected the distribution of each particular mode among the DEGs, then compared this distribution with those across all genes detected in nodules. While we observed no statistically significant difference between the distribution of WGD, PD, and RD duplicates, TD and DSD duplicates were significantly overrepresented in DEGs and non-duplicated genes were significantly underrepresented in DEGs. The results showed the non-random distribution of a particular mode in DEGs and the preferential representation of duplicated genes connected with BNF efficiency. According to Qiao et al. [106], TD together with PD showed no significant decrease in frequency over time, thus indicating that this mode of duplication offers a continuous supply of genetic material for evolution and important genetic material for rapidly changing environments [155]. Dispersed duplicates are among the most prevalent duplication modes in genomes across different plant species [156]. Expression divergence analysis showed that about 75–80% of duplicated gene pairs diverged from each other in all those duplication modes analyzed, but the answer as to why only TDs and DSDs are overrepresented in DEGs remains unknown.

WGCNA analysis complements DEGs analysis and enables the arrangement of other transcripts. WGCNA analysis is used to classify genes according to their expression profiles. Genes with similar expression patterns may form clusters (modules) [157]. Transcripts in one module have a similar transcription pattern through all RNA-seq samples. In terms of nitrogen fixation, modules Blue, Red, and Salmon are negatively correlated and Greenyellow, Turquoise, and Purple are positively correlated. Of 491 DEGs, 51% belong to the Blue module and 15%,14%, 8%, and 7%, respectively, to the Turquoise, Brown, Red and Yellow modules. The remaining DEGs are spread across other modules or were filtered prior to WGCNA analysis. Among putative NCR genes, 3 (gene18074, gene23764, and gene38999) of 8 such genes were part of the Blue module and 1 (gene33781) was part of the Turquoise module. Another 4 identified NCR genes do not fulfill wgcna filter criteria for minimal expression level and expression variance across samples.

An interesting question is of where the known core genes of the root symbiotic nitrogen process appear. To answer this, we borrowed a list of 19 core predisposition genes that were collected in other studies within closely related species, in particular *M. truncatula* [158]. We found their red clover orthologues and then their localization in the WGCNA network and in the DEGs list (Supplementary Table S6). Fifteen core genes are captured in the most common Turquoise module, 2 core genes in the Brown module, and 1 each in the Blue, Green, Greenyellow, and Yellow modules. 

Interestingly, no core gene was identified among the DEGs, indicating that the differential phenotype of nitrogen fixation levels is realized not at the level of symbiosis establishment and symbiotic structure formation but rather at the level of fixation regulation. This is supported by the fact that we are comparing not zero fixation level with non-zero but lower with higher fixation levels.

Nitrogen fixation through root nodule symbiosis is an essential process by which diazotrophic organisms make otherwise unavailable nitrogen available for their life needs and, through themselves, make it available to other living organisms. The phenomenon of symbiotic nitrogen fixation has evolved multiple times independently in one evolutionary branch of angiosperms that has been termed the “Nitrogen-fixing clade”. We can assume that, prior to the actual development of the ability to fix nitrogen, plants of this clade must have been predisposed through a support mechanism already in place [158,159]. It is probable that a broad and very complex transcriptomic background allowed nitrogen fixation to evolve while enabling the preservation of transcriptomic diversity in fixing nodules. 

In red clover, an important non-model plant and forage crop, we found 491 differentially expressed genes connected with BNF efficiency. Subsequent annotation of genes in nodule transcriptome revealed more than 800 genes not yet experimentally confirmed. We were able to confirm only four nodule-specific cysteine-rich (NCR) peptides in the nodule transcriptome. In addition, we found unequal distribution of different modes of gene duplication in DEGs, with genes originating from tandem and dispersed duplication being significantly overrepresented in DEGs. Finally, using WGCNA we organized expression profiles of the transcripts into 16 modules linked to the analyzed traits, such as nitrogen fixation efficiency or sample-specific modules. Nodule transcriptomics is a rewarding topic. A series of transcriptomic studies have revealed transcripts associated with the root nodule symbiotic process [15,160,161,162,163,164,165,166,167,168,169,170,171,172,173]. The DEGs identified in this study and their analyses allowed a comparison to the nodule transcriptome in genotypes with different BNF efficiency and provided a valuable resource for further investigation of the genetic basis of this trait of interest.

## Figures and Tables

**Table 1:** Top 10 pathways with highest numbers of enzymes encoded by DEGs.

Pathway	Pathway ID	Enzymes in Pathway
Sesquiterpenoid and triterpenoid biosynthesis	map00909	12
Pentose and glucuronate interconversions	map00040	6
Pantothenate and CoA biosynthesis	map00770	5
Steroid hormone biosynthesis	map00140	5
Glycerolipid metabolism	map00561	5
Galactose metabolism	map00052	4
Amino sugar and nucleotide sugar metabolism	map00520	4
Cysteine and methionine metabolism	map00270	4
Starch and sucrose metabolism	map00500	4
Phenylpropanoid biosynthesis	map00940	4

**Table 2:** Confirmed NCR peptides detected in nodules of *T. pratense* using RNA sequencing. *Padj*-value < 0.05 indicates differentially expressed gene.

Sequence ID	BLAST	Domains	Localization	*Padj*-Value
Tp57577_TGAC_v2_gene38999	Defensin-like protein	Gamma-thionin family, Knot1, Knottin fold	*p* = 0.9999 − Extracellular	1.33 × 10^−8^
Tp57577_TGAC_v2_gene36456	Defensin-like protein	Knot1	*p* = 0.9993 − Extracellular	0.133
Tp57577_TGAC_v2_gene7879	Defensin-like protein		*p* = 1 − Extracellular	0.894
Tp57577_TGAC_v2_gene30230	Defensin-like protein		*p* = 1 − Extracellular	0.716

**Table 3:** Distribution of different modes of duplication across differentially expressed genes (DEGs) and genes expressed in nodules (EG) with *p*-values.

Duplication Mode	DEG (%)	EG (%)	*p*-Value *
WGD	3.96	4.03	1
Tandem	14.58	9.91	0.0012
Proximal	5.21	5.34	1
Transposed	27.71	30.89	0.1358
Dispersed	37.08	32.75	0.0447
Non-duplicated	11.46	17.08	0.0008
All	100.00	100.00	

WGD–whole genome duplication, * two-tailed Fisher’s exact test.

**Table 4:** Frequency of genes in modules.

Module Colors	Genes Frequency
Turquoise	9097
Blue	7178
Brown	3540
Yellow	1635
Green	1488
Red	712
Black	486
Pink	486
Magenta	329
Purple	210
Greenyellow	174
Tan	158
Salmon	153
Cyan	146
Grey	50
Midnightblue	31

**Table 5:** Top 10 enriched GO bp terms.

GO ID	GO Name	*p*-Value
GO:0051704	multi-organism process	3.56 × 10^−35^
GO:0006950	response to stress	1.41 × 10^−29^
GO:0050896	response to stimulus	2.52 × 10^−29^
GO:0009605	response to external stimulus	1.74 × 10^−27^
GO:0001101	response to acid chemical	1.79 × 10^−26^
GO:0048856	anatomical structure development	8.30 × 10^−26^
GO:0042221	response to chemical	2.04 × 10^−25^
GO:0032502	developmental process	8.03 × 10^−25^
GO:0009607	response to biotic stimulus	2.16 × 10^−24^
GO:0043207	response to external biotic stimulus	3.40 × 10^−23^

**Table 6:** Top 10 genes with the highest expression in nodules. Mean expression is the number of reads assigned to a particular gene. This number was divided by the length of the sequence for normalization to different sequence lengths.

Gene ID	Annotation	Mean Expression
Tp57577_TGAC_v2_gene25864	Leghaemoglobin	418.76
Tp57577_TGAC_v2_gene23575	Nodulin	210.49
Tp57577_TGAC_v2_gene4062	Leghaemoglobin	180.215
Tp57577_TGAC_v2_gene25862	Leghaemoglobin	127.54
Tp57577_TGAC_v2_gene25853	Leghaemoglobin	60.69
Tp57577_TGAC_v2_gene23582	IBR protein/transcription factor	59.54
Tp57577_TGAC_v2_gene39897	Nodulin/Aquaporin	55.20
Tp57577_TGAC_v2_gene608	Embryo-specific protein	51.20
Tp57577_TGAC_v2_gene29022	Asparagine synthetase	43.71
Tp57577_TGAC_v2_gene25837	Leghaemoglobin	43.10

## Data Availability

Data is contained within the article and Supplementary Materials.

## Supplementary Materials

The following supporting information can be downloaded at: https://www.mdpi.com/article/10.3390/life12121975/s1, Supplementary Figure S1: Quality control of the mapped reads. Supplementary Figure S2: Example of Pearson correlation coefficient of biological replicates. Supplementary Figure S3: Dendrogram of rlog-transformed read counts. Supplementary Figure S4: PCA plot of rlog-transformed read counts. Supplementary Figure S5: MA plot of genes detected in nodules. Supplementary Figure S6: Eigengenes expression profiles of 16 WGCNA modules. Supplementary Figure S7: WGCNA network. Supplementary Figure S8: Heatmap of relationships between traits and module eigengenes. Supplementary Table S1: Primer pairs used for qPCR verification of sequencing data. Supplementary Table S2: List of differentially expressed genes between genotypes with high and low BNF efficiency. Supplementary Table S3: Expression distribution of originally unconfirmed genes detected in nodules. Supplementary Table S4: Top 10 GO bp terms in originally unconfirmed genes detected in modules. Supplementary Table S5: List of originally unconfirmed genes. Supplementary Table S6: Annotation summary of GO terms for trait-correlated WGCNA modules and DEGs.

## Abbreviations

ARA	Acetylene reduction assay
ASNSM	Amino sugar and nucleotide sugar metabolism
BNF	Biological nitrogen fixation
CRISPR	Clustered Regularly Interspaced Short Palindromic Repeats
Ct	Cycle threshold
DEG	Differentially expressed gene
DSD	Dispersed duplication
FDR	False discovery rate
GM	Galactose metabolism
GO	Gene Ontology
KEGG	Kyoto Encyclopedia of Genes and Genomes
KO	Kegg orthology
KOG	Clusters of Orthologous Groups
NCR	Nodule-specific cysteine-rich
*padj*	adjusted *p*-value
PCA	Principal component analysis
PD	Proximal duplication
PGI	Pentose and glucuronate interconversions
RD	Transposed duplication
qPCR	quantitative polymerase chain reaction
RNAi	RNA interference
SSM	Starch and sucrose metabolism
TD	Tandem duplication
Tp	Trifolium pratense
WGCNA	Weighted correlation network analysis
WGD	whole genome duplication
