# Supplementary material for: Genes Associated with Biological Nitrogen Fixation Efficiency Identified Using RNA Sequencing in Red Clover (Trifolium pratense L.)
Source: Life (Basel). 2022 Nov 25;12(12):1975. doi: 10.3390/life12121975 (PMC9785344; doi:10.3390/life12121975)
Supplement: Supplementary file 1 [file life-12-01975-s001.zip › Supplementary_figures.pptx]

## Slide 1
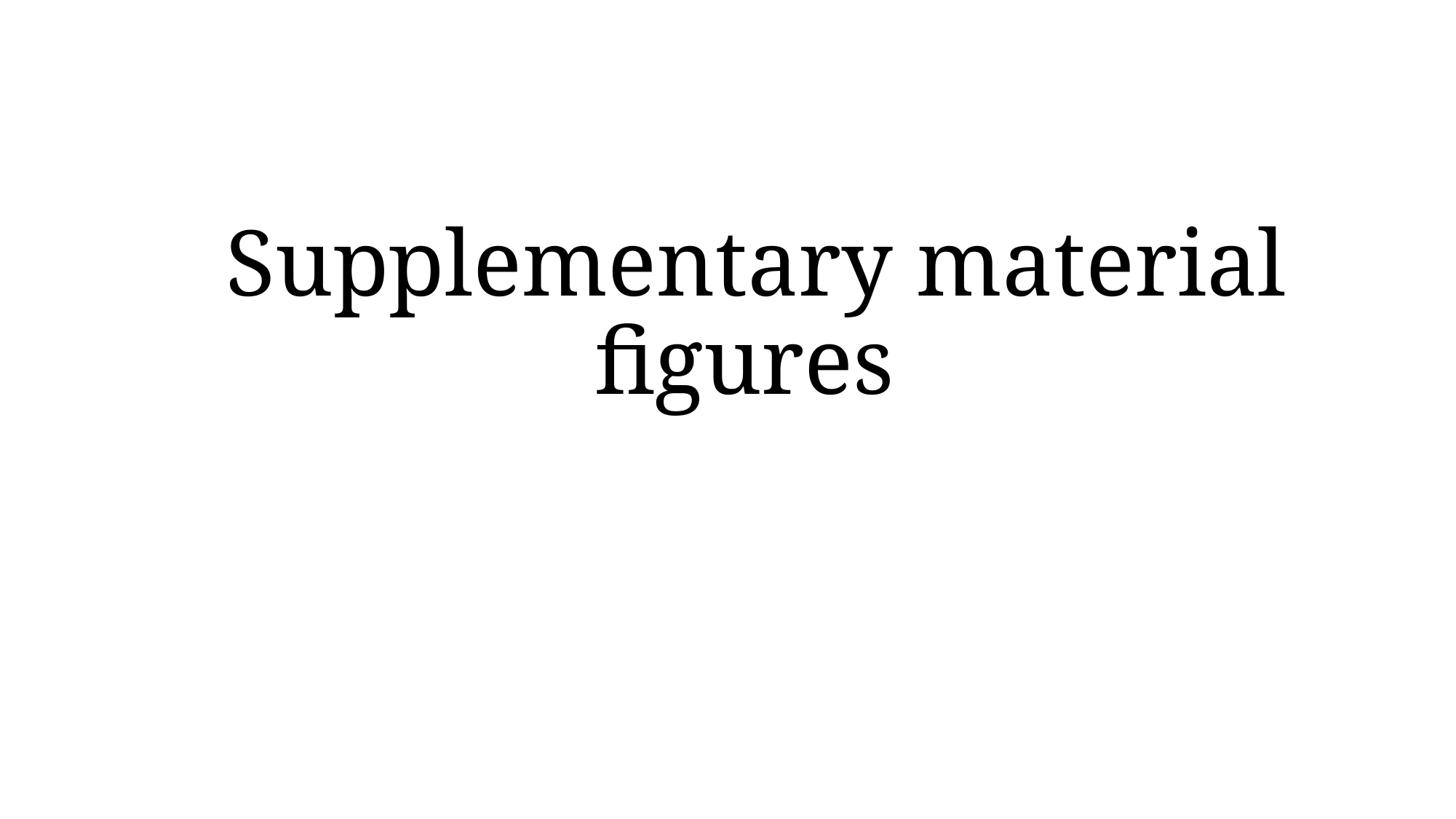

# Supplementary material figures

## Slide 2
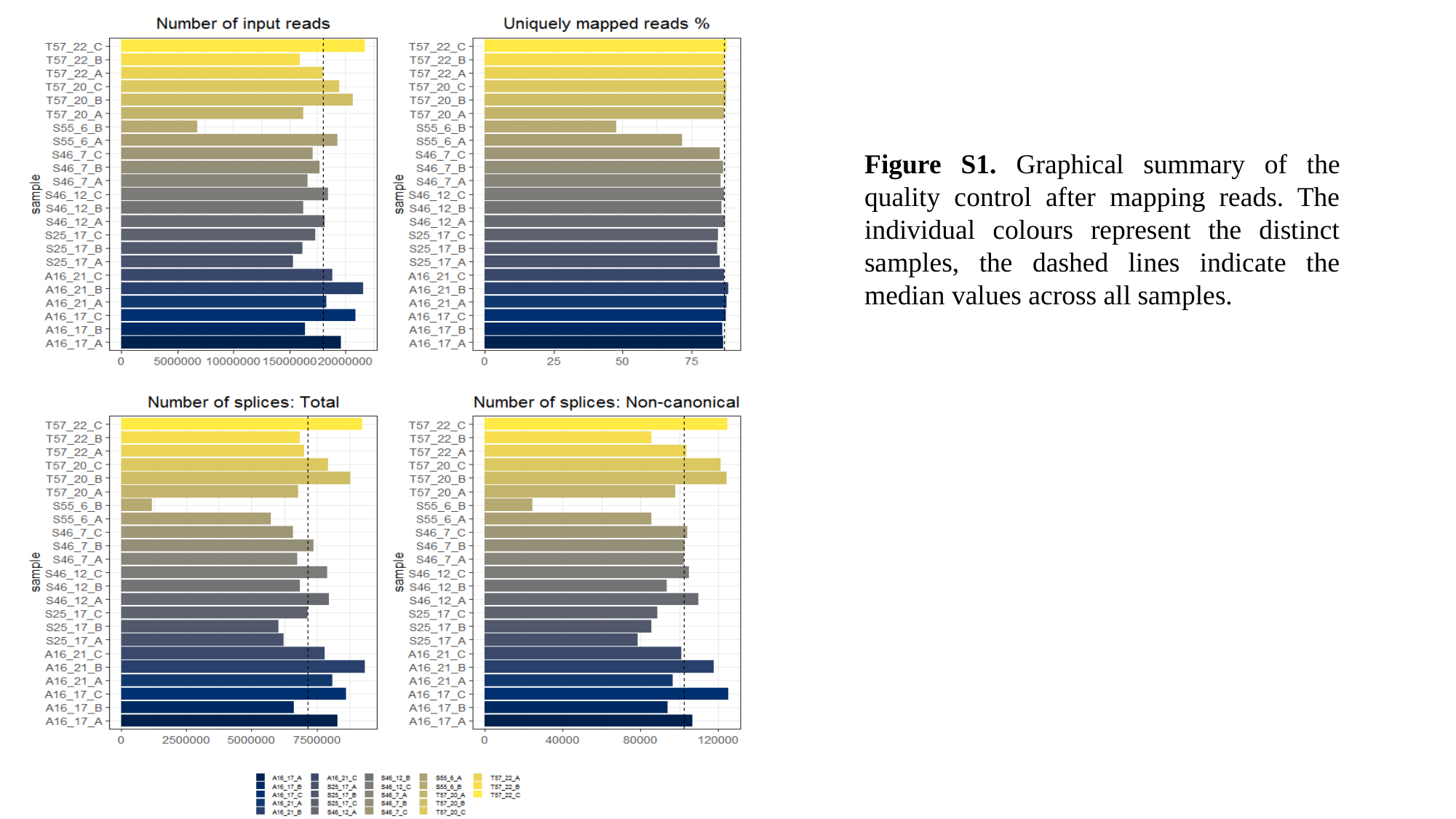

Figure S1. Graphical summary of the quality control after mapping reads. The individual colours represent the distinct samples, the dashed lines indicate the median values across all samples.

## Slide 3
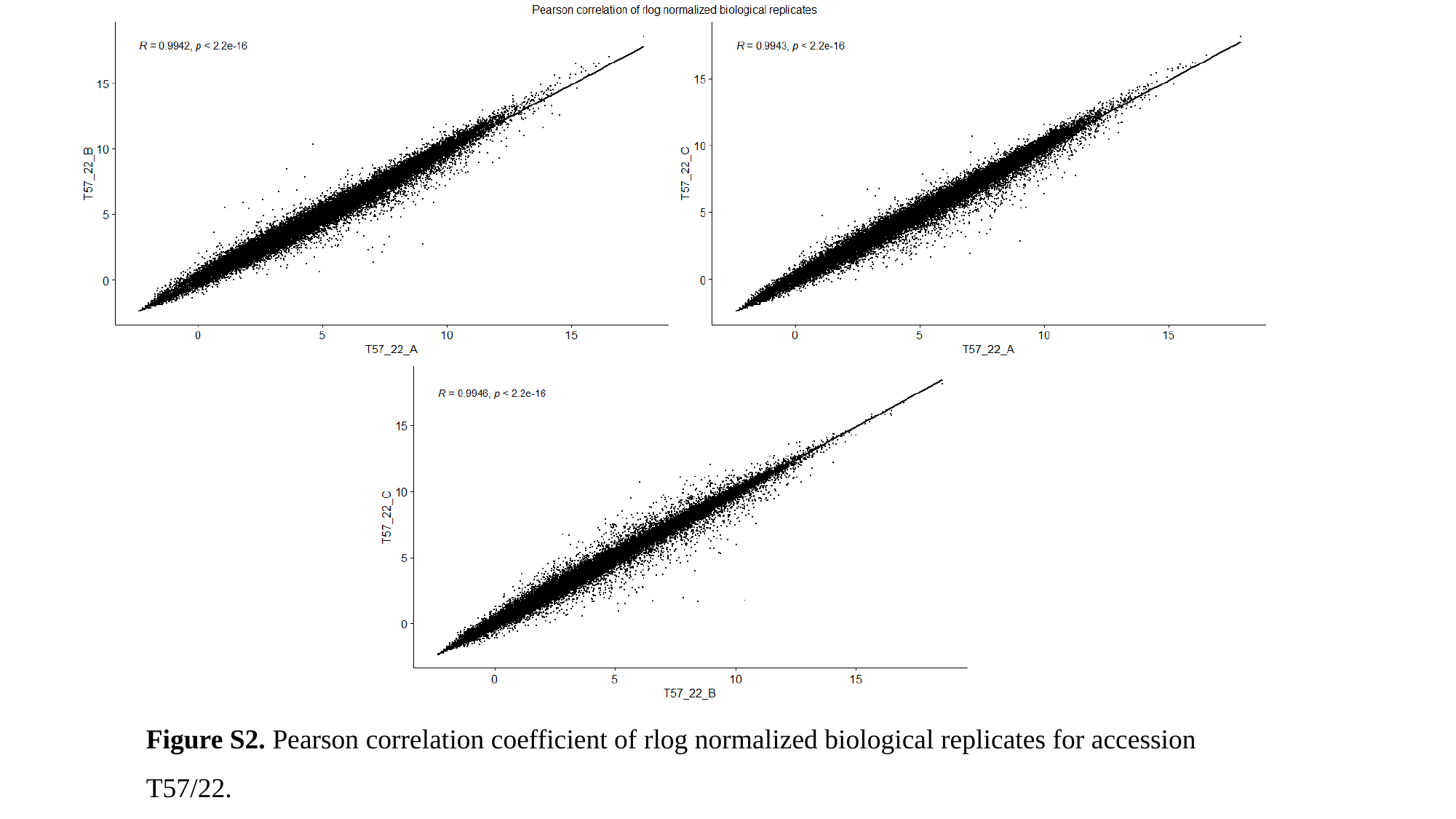

Figure S2. Pearson correlation coefficient of rlog normalized biological replicates for accession T57/22.

## Slide 4
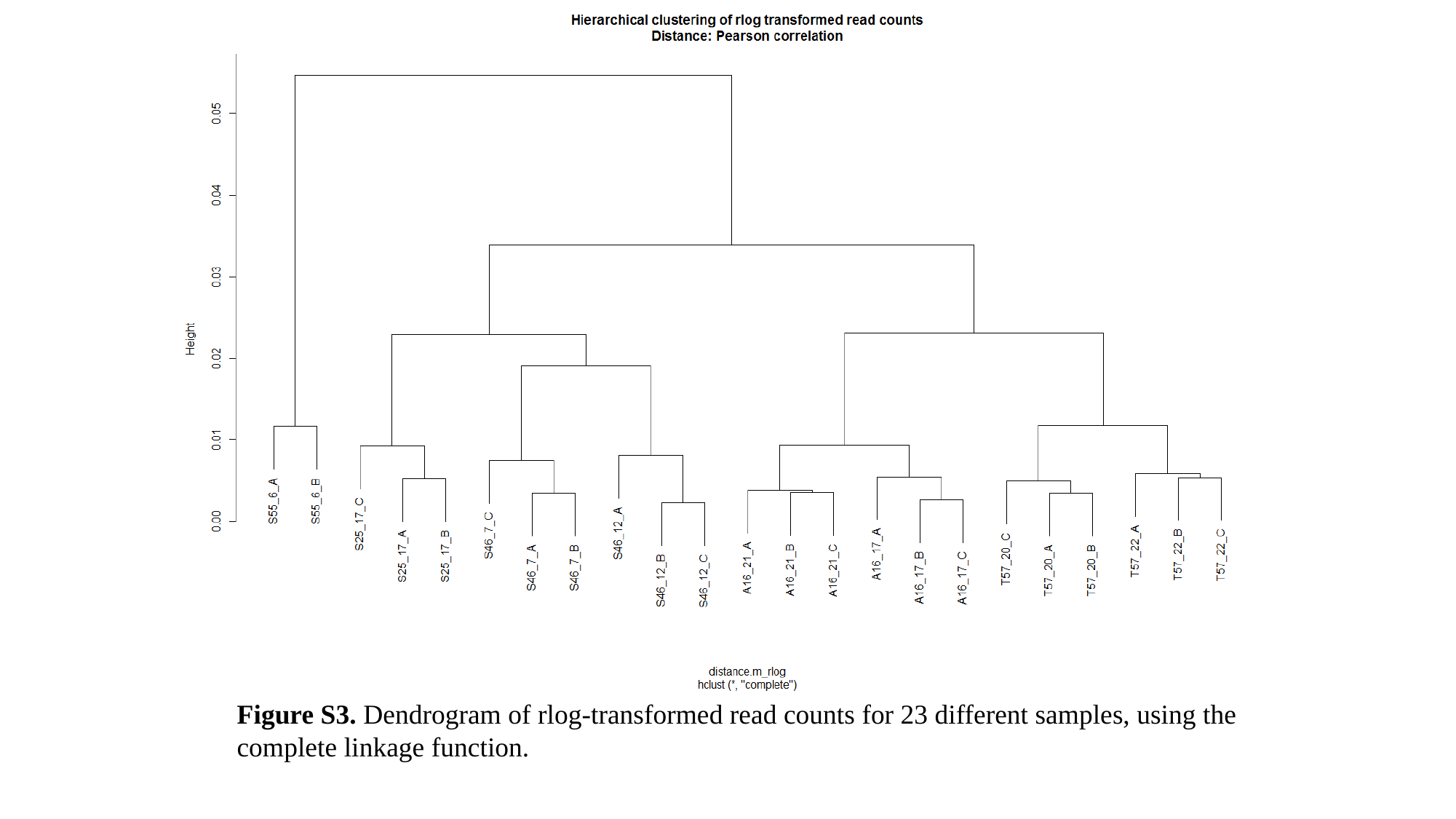

Figure S3. Dendrogram of rlog-transformed read counts for 23 different samples, using the complete linkage function.

## Slide 5
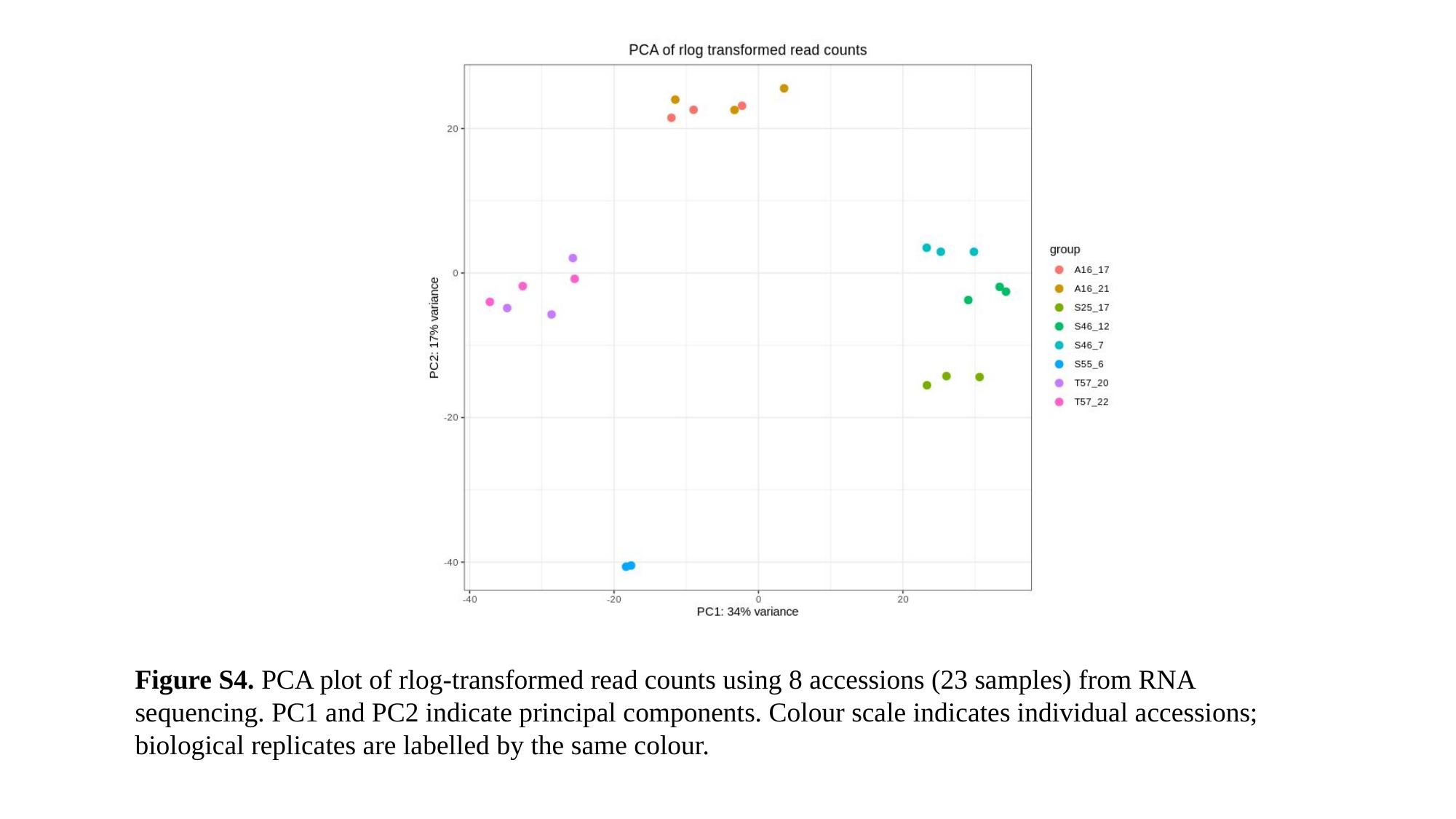

Figure S4. PCA plot of rlog-transformed read counts using 8 accessions (23 samples) from RNA sequencing. PC1 and PC2 indicate principal components. Colour scale indicates individual accessions; biological replicates are labelled by the same colour.

## Slide 6
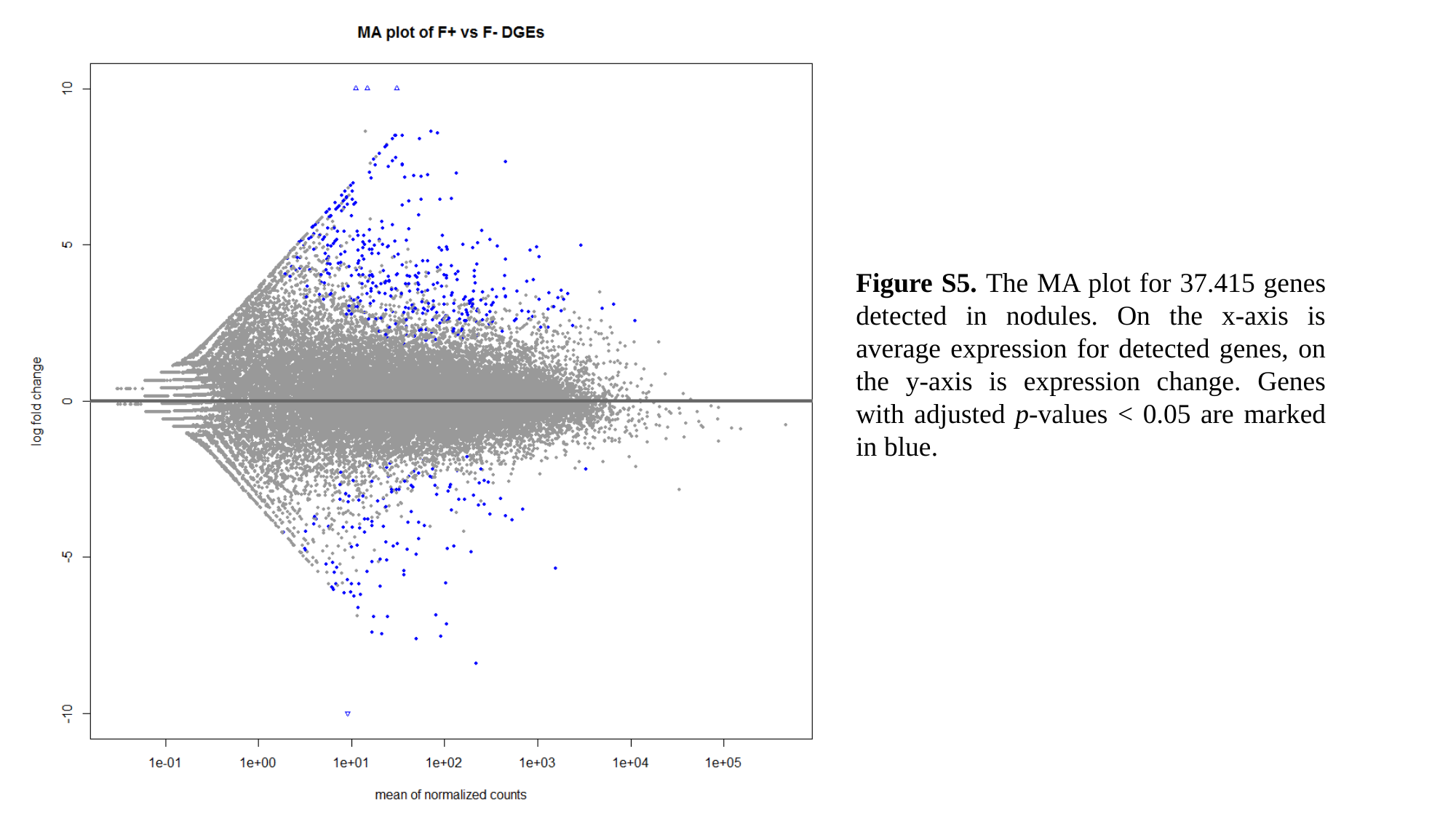

Figure S5. The MA plot for 37.415 genes detected in nodules. On the x-axis is average expression for detected genes, on the y-axis is expression change. Genes with adjusted p-values < 0.05 are marked in blue.

## Slide 7
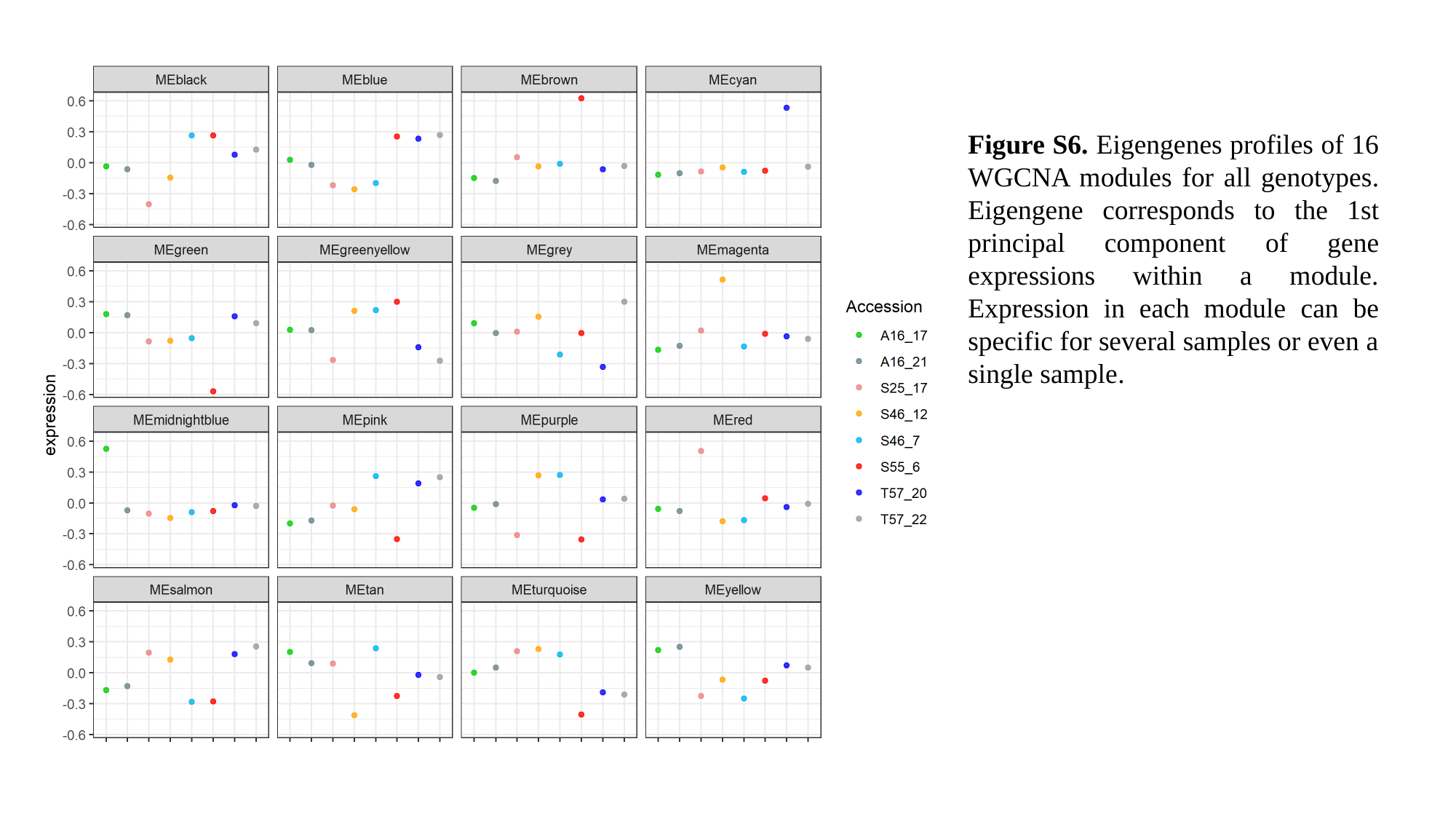

Figure S6. Eigengenes profiles of 16 WGCNA modules for all genotypes. Eigengene corresponds to the 1st principal component of gene expressions within a module. Expression in each module can be specific for several samples or even a single sample.

## Slide 8
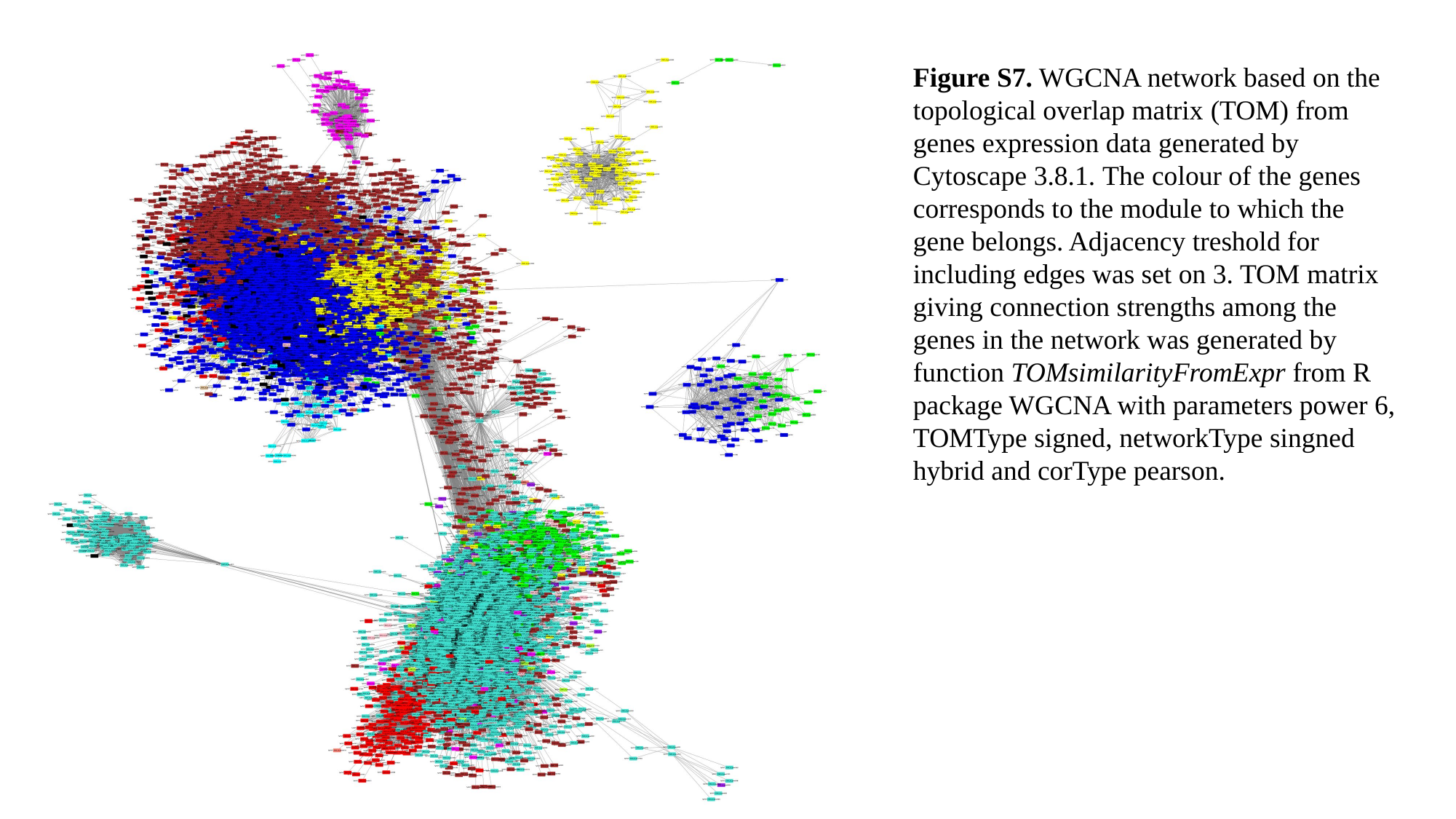

Figure S7. WGCNA network based on the topological overlap matrix (TOM) from genes expression data generated by Cytoscape 3.8.1. The colour of the genes corresponds to the module to which the gene belongs. Adjacency treshold for including edges was set on 3. TOM matrix giving connection strengths among the genes in the network was generated by function TOMsimilarityFromExpr from R package WGCNA with parameters power 6, TOMType signed, networkType singned hybrid and corType pearson.

## Slide 9
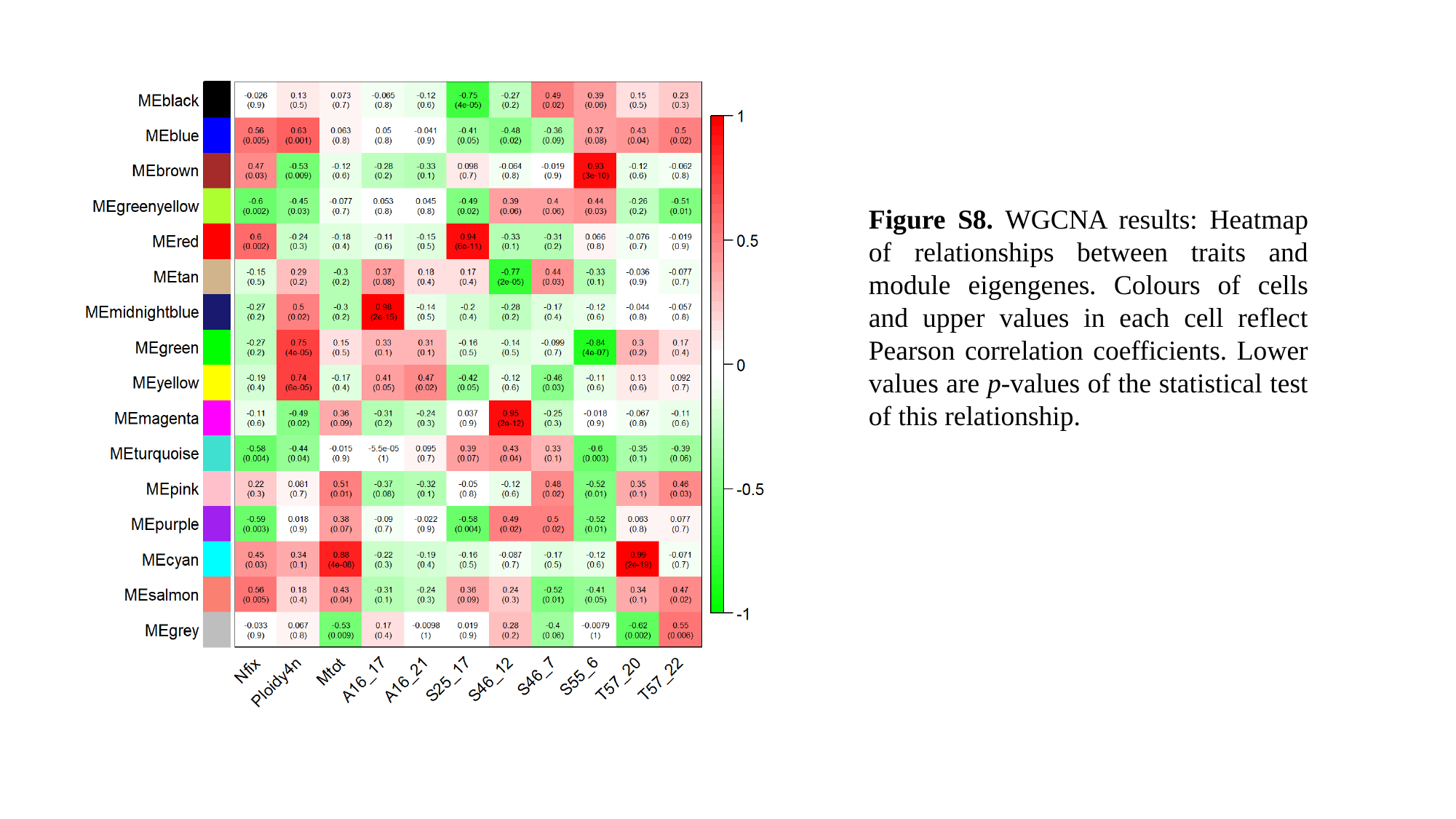

Figure S8. WGCNA results: Heatmap of relationships between traits and module eigengenes. Colours of cells and upper values in each cell reflect Pearson correlation coefficients. Lower values are p-values of the statistical test of this relationship.
